# Novel telemetric pressure monitoring in lumbar theca

**DOI:** 10.1016/j.bas.2022.100886

**Published:** 2022-03-31

**Authors:** Sogha Khawari, Kanza Tariq, Lucia Darie, Eleanor Moncur, Ahmed Toma, Laurence Watkins

**Affiliations:** Victor Horsley Department of Neurosurgery, National Hospital for Neurology and Neurosurgery, London, UK

**Keywords:** Lumboperitoneal shunt, Telemetric sensor, Telesensor, Thecal pressure, Cerebrospinal fluid pressure

## Abstract

**Introduction:**

There is no previous literature on the use of telemetric sensors (telesensor) in the lumbar theca. We aim to provide novel data on telemetric pressure monitoring of the lumbar theca via lumboperitoneal shunts.

**Research question:**

Primary outcome is telemetric sensor malfunction of lumboperitoneal shunt. The secondary outcome is post-operative complications.

**Materials and methods:**

A single centre retrospective case series of patients with telemetric sensor in LP shunt system, between 2015 and 2021, consisting of 5 patients. Review of indications for use, duration of function of telemetric sensor and associated complications.

**Results:**

There was no procedural complications of LP shunt insertion with telemetric sensor. The patient with highest body weight patient had retraction of distal tubing which required distal resiting 3 times. Four out of five patients had no complications. In all cases, telemetric sensor functioned satisfactorily with no dysfunction. The duration of documentation was 1–40 months. Pressure readings were satisfactorily carried out in variety of positions.

**Discussion and conclusion:**

This is the first report of telemetric sensor use in the lumbar theca. It can provide a valuable way of measuring cerebrospinal fluid pressures, particularly in patients avoiding cranial surgery. More research is indicated to assess what pressure values would mean clinically.

## Highlights

• Technical note on novel telemetric sensors in the lumbar theca.
• Study shows excellent function of telemetric sensors in the lumbar theca with no dysfunction.
• We encourage the use of telemetric sensors in lumbar theca to allow for further data and interpretation of CSF pressures.

## 1 Introduction

Hydrocephalus is defined as dysfunction of cerebrospinal fluid (CSF) dynamics with many causes. CSF diversion procedures can be performed in various ways. An established management option of communicating hydrocephalus is a lumboperitoneal (LP) shunt. Although LP shunts have been largely phased out by ventriculoperitoneal (VP) shunts, scope for LP shunts still exist in certain circumstances (WangM.D. et al., 2007). Indication can be idiopathic intracranial hypertension (IIH), hydrocephalus post brain haemorrhage and normal pressure hydrocephalus. The indication for LP shunts for communicating hydrocephalus are widening globally. The advantage of LP shunt over VP shunt are reported as minimal brain injury and low incidence of postoperative infection (WangM.D. et al., 2007). Patient choice should also be reviewed, particularly to reduce intracranial complications, driving and occupation restrictions which accompanies cranial surgeries. The study by Azad et al. (n ​= ​1082) suggest that LP and VP shunts, used in IIH, have comparable rates of shunt failure and complications. Revision rate did not differ significantly between VP and LP shunt (24.6% VS 23.9%) (Tej D Azad et al., 2020). On the contrary, Menger et al. (n ​= ​4480), showed revision rates were higher in the LP shunt sample (7% vs 3.9%). Limitation of this study was the lack of indications of one shunt over the other, and the inability to follow a single patient, therefore readmission of the same patient is not cumulative (Menger et al., 2014). The open-label randomised trial, SINPHONI-2, showed that LP shunt insertion resulted in improvement of one point or more on the modified Rankin Scale at 3 months in patients with Normal Pressure Hydrocephalus (NPH) (Kazui et al., 2015). A randomised controlled trial is currently underway to compare VP and LP shunt for NPH which could provide level I evidence (Cui et al., 2021).

Telemetric VP shunting systems have been used since the late 1970s (A telemetric pressure sen, 1979). The fully implantable telemetric sensor is incorporated within the shunt valve system and provides novel non-invasive method of measuring intraventricular pressure. Despite numerous publications on cranial telemetric sensor, there is no literature on telemetric sensor use in LP shunts. From previous literature, a difference of pressure readings from lumbar puncture in lateral decubitus was noted from intracranial pressure via intraparenchymal ICP monitoring (D'Antona et al., 2019). Further changes in pressure are also noted in different positions, such as neck flexion and hip flexion (Pedersen et al., 2021).

This series is a novel study to assessing telemetric pressure monitoring in the lumbar theca. It offers paramount importance as the hardware of the LP systems currently offer very little possibilities to check for patency and pressure. The telemetry is based on a reliable system and is easily applicable to commercially available LP systems. The series focuses on evaluating its duration of function, complications, and thecal pressure readings in different positions.

## 2 Research Question

The primary outcome of this case series is telemetric sensor malfunction of LP shunt. The secondary outcome is post-operative complication of LP shunt with telemetric sensor insertion. This included general post-operative complications including infection, bleeding, disability, as well as shunt malfunctions such as shunt blockage, retraction, and misplacement. Time to shunt failure was recorded as number of months from insertion. Duration of shunt being in situ was recorded as number of months. We hypothesised satisfactory telemetric sensor function in patients with LP shunt.

## 3 Materials and Methods

Literature search was carried out via Embase and Ovid Medline (R) ALL which revealed no articles on lumbar thecal telesensor. The search for “telemetric” or “telesensor” revealed 5046 articles, the search for “thecal” or “lumboperitoneal” returned 6365 articles, however no articles were found when these 2 groups of searches were combined.

A single centre case series with retrospective analysis of medical notes of patients with LP shunt with telemetric sensor between 2015 and 2021. Demographic variables were collected including gender, age at insertion and indication for shunt placement. Head and lumbar imaging was analysed. Notes were reviewed to gather any pressure readings using telemetric sensor of LP shunt, including the position pressure was read in. Pressure readings from clinic or inpatient stay was used. Patients were above 18 years old. No patients were excluded on basis of indication for shunt.

Shunt system used consisted of a Miethke Sensor Reservoir, M. Scio (Miethke GmbH, Potsdam, Germany) and Strata valve (Medtronic, Minneapolis, USA) inserted as part of a LP shunt system.

## 4 Results

**Fig. 1:**
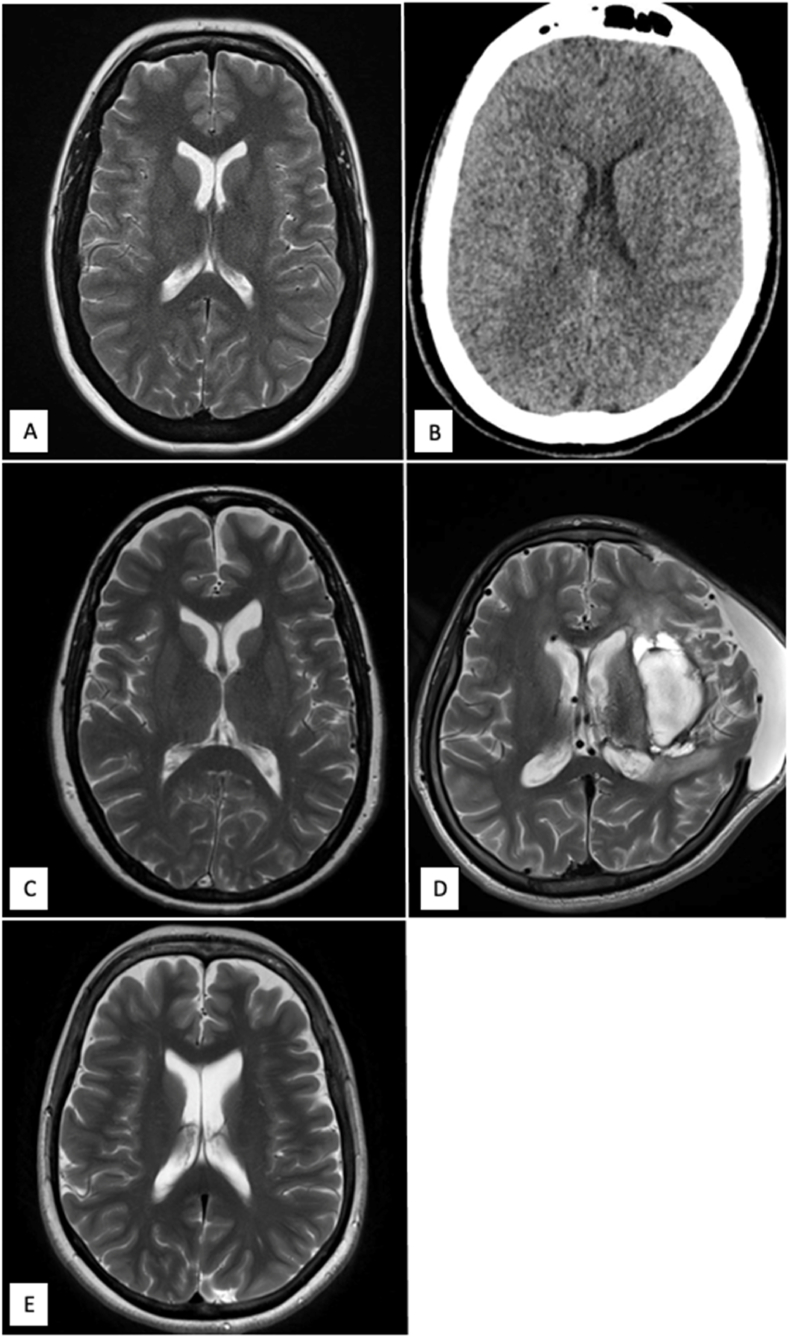
Head imaging of all patients (A to E), axial MRI or CT head. Mostly no gross abnormality, except patient D who had a previous bleed from an arteriovenous malformation with a decompressive craniectomy.

**Fig. 2:**
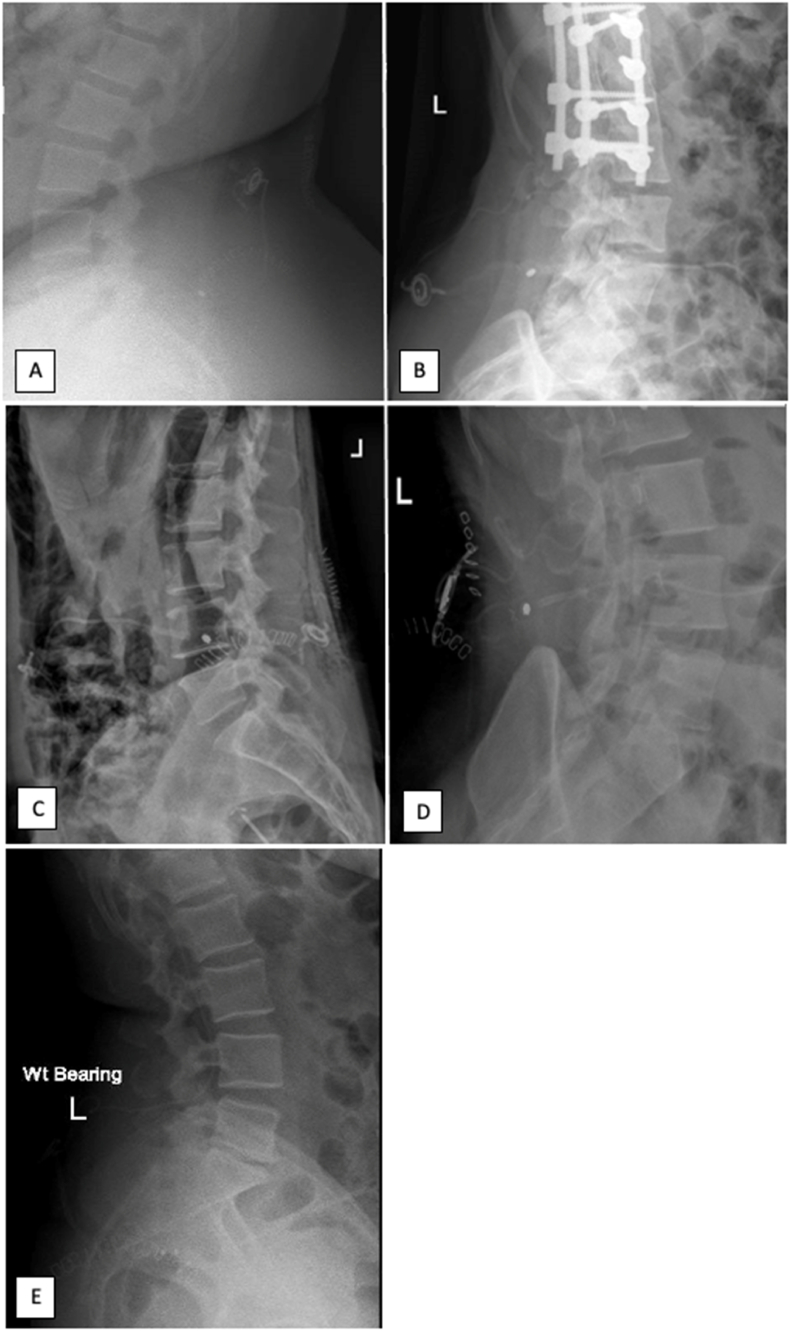
Lumbar radiographs of patients A to E, showing lumboperitoneal shunt consisting of telemetric sensor and adjustable valve.

Retrospective analysis revealed a total of 5 patients with telemetric sensor in the lumbar theca. Four out of five patients were female. Age range was 21–51 years of age at time of insertion. Indication was mainly for patient preference of treatment of IIH. Head and lumbar imaging of all patients are shown in Fig. 1, Fig. 2. In one patient, LP shunt was chosen over VP shunt to reduce risk of bleeding from AVM. Pressure readings were carried out in a range of positions, consisting of standing, supine, lateral decubitus and sitting position. Results ranged from −2 to 49 ​mmHg. Duration of telemetric sensor were all ongoing with no malfunction, maximum recorded is 40 months, as seen in Table 1.

**Table 1:** Patient demographics and shunt duration.

	Sex	Age at insertion (yrs)	Weight (kg)	Indication for LP shunt	Shunt malfunction or complications	Total duration of telesensor use (months)
A	F	34	113 121 138	IIH and patient preference	Distal end revisions x3 - 3months - 10months - 46months	32
B	F	21	83	IIH	Nil	14
C	F	51	63	IIH	Nil	40
D	M	21	59	Hydrocephalus due to ICH	Nil	1
E	F	48	82	Hydrocephalus	Nil	1

Only one patient had post-operative complication which was shunt retraction occurring three times at 3 months, 10 months, and 46 months. A pattern of shunt retraction was seen which is weight gain at each of the shunt retraction episodes. Patient underwent distal end resiting each time.

None of the patients had any complications of the telemetric sensor and all were able to have reading of their CSF pressure in the outpatient clinic over subsequent months, as seen in Table 2. The duration from implantation to the most recent recorded pressure measurement from the telemetric sensor ranged from 1 to 40 months.

**Table 2:** Lumbar thecal pressure readings in different positions.

	Time after last surgery (months)	Valve settings	Standing Pressure (mmHg)	Supine Pressure (mmHg)	Lat decubitus (mmHg)	Sitting position (mmHg)
A	1 19 12	2.5 2.5 2.0	32 37	22 27	12.5	25
B	0 5 14	2.5 2.5 2.5	49 35	14 29 21		
C	5 40	2.5 2.5		58	27	30
D	1	0.5	14	10		11
E	1	2.5			9.8	

### 4.1 Patient A

Patient A is a 39-year-old female with idiopathic intracranial hypertension. She previously required a right transverse sinus stent and optic nerve fenestration, the former thrombosed and the latter was unsuccessful. The patient was admitted with worsening vision. Ophthalmology assessment revealed bilateral papilloedema. Intracranial pressure monitoring demonstrated raised intracranial pressure. The patient was hesitant to accept a VP shunt, therefore a LP shunt with a Medtronic Strata valve with Sensor Reservoir was inserted at this stage. There were no intra- or immediately post-operative complications. Three months after insertion, the patient presented with soft abdominal lump. This was found to be a pseudomeningocele caused by retraction of distal tubing. Patient reported recent weight gain, her weight at the time was 113 ​kg with a body mass index of 41. Patient underwent drainage of pseudo-meningocele and revision of distal tubing. Almost a year later, patient reported abdominal discomfort with no signs of infection. At this stage, the LP shunt was externalized and re-introduced at a later stage once symptoms settled.

Pressure readings were carried out in outpatient clinic over the years. First outpatient readings were performed 1 month after inserted, lateral decubitus position was 12.5 ​mmHg and sitting position of 25 ​mmHg. Two years later, on a valve setting of 2.5, standing pressure was 32 ​mmHg and supine was 22 ​mmHg. Four years after insertion of LP shunt, patient represented with an abdominal lump. Further imaging showed a repeated pseudomeningocele with distal shunt retraction. Her weight at this time had increased to 138 ​kg. She underwent distal shunt tubing revision. In summary, this patient had a LP shunt in situ for 40 months, with three distal end revisions but otherwise no other shunt complications, in particular no telemetric sensor malfunction. Clinic follow up thereafter, showed standing pressure of 37 ​mmHg and supine of 27 ​mmHg, on a valve setting of 2.0.

### 4.2 Patient B

Patient B is a 22-year-old female with a first diagnosis of idiopathic intracranial hypertension. She presented locally with a one-month history of headaches, blurred vision and vomiting. Her weight at presentation was 83 ​kg. Lumbar puncture opening pressure was 70 ​mmHg and ophthalmology team reported bilateral papilloedema. Patient was transferred urgently to our centre. Patient underwent ICP monitoring with an opening pressure of 63 ​mmHg, median ICP of 22 ​mmHg and median pulse amplitude of 8 ​mmHg. Venous imaging showed focal stenosis between the lower transverse and sigmoid sinus. Venous stenting of the transverse-sigmoid junction was carried out. Thereafter ophthalmology review revealed ongoing grade 4 haemorrhagic papilloedema bilaterally. Further ICP monitoring post stent insertion showed ongoing high intracranial pressures, with median ICP of 30 ​mmHg. Repeat venous imaging confirmed no stent thrombosis. Patient required immediate cerebrospinal fluid drainage to protect her vision, thus underwent LP shunt insertion with Sensor Reservoir and Medtronic Strata valve and telemetric sensor, set at 2.5. There were no intra- or post-operative complications. Patient improved clinically and was discharged.

Clinic follow-up five months after insertion, showed pressures of 49 ​mmHg in standing position and 29 ​mmHg in supine position. Ophthalmology assessment was carried out which revealed no disc swelling and visible SVPs. Further pressure readings were carried out in follow-up fourteen months post insertion of shunt; standing pressure of 35 ​mmHg and supine pressure of 21 ​mmHg. In summary, lumbo-peritoneal shunt functioning well at 14 months with no shunt malfunction or post-operative complications.

### 4.3 Patient C

Patient C is a 54-year-old female who presented with worsening visual symptoms on a background of idiopathic intracranial hypertension. Her weight at presentation was 63 ​kg. Intracranial pressure monitoring was carried out showing raised pressures. Patient underwent insertion of LP shunt with Medtronic Strata valve, set to 2.5, with Sensor Reservoir. There were no intra- or post-operative complications. Pressure readings at 5 months post insertion was 30 ​mmHg in sitting position, readings at 40 months were 58 ​mmHg in supine position and 27 ​mmHg in lateral decubitus position. To conclude, a LP shunt with no shunt malfunction or post-operative complications at 40 months post insertion.

### 4.4 Patient D

Patient D is a 22-year-old male who presented as an emergency admission with intracerebral haemorrhage due to a left temporal arteriovenous malformation. He underwent hemicraniectomy with later cranioplasty insertion. Thereafter he developed worsening pseudomeningocele overlying the cranioplasty. Due to risk of AVM bleed from ventriculoperitoneal shunt insertion, best interest decision was made to proceed to a LP shunt insertion. Medtronic Strata valve set at 0.5 with Sensor Reservoir was used. Pressure readings were carried out at 1 month after insertion, showed 14 ​mmHg, 10 ​mmHg and 11 ​mmHg in standing, supine and sitting position respectively.

### 4.5 Patient E

Patient E is a 48-year-old female who presented with 10-year history of progressive gait disturbance and visual symptoms. Previous lumbar puncture carried out elsewhere had shown raised opening pressure with good response to large volume drainage. Head imaging was unremarkable. ICP monitoring revealed a median ICP of 7.8 ​mmHg with a pulse amplitude of 5.7, with a peak ICP reading of 55 ​mmHg. The moderately raised intracranial pressure and abnormal CSF compliance suggested a possible degree of intracranial hypertension. Management options were discussed, and patient preference was lumboperitoneal shunt insertion to avoid cranial surgery. Patient underwent insertion of LP shunt with Medtronic Strata valve and telemetric sensor. Patient was reviewed 4 weeks post-surgery, with improvement in her headache and visual symptoms. Pressure readings in the left lateral position was noted to be 9.8 ​mmHg with a good waveform.

## 5 Discussion and Conclusion

This technical note based on a case series aimed to review function of telemetric sensor in the lumbar theca, as part of an LP shunts. No telemetric sensor dysfunction was noted, and thecal pressures were able to be monitored up to at least 40 months. There was 1 post-operative complication consisting of distal shunt tubing retraction in the patient with the highest BMI. Potential hypothesis for this would be increase in abdominal mass causes increase in kinetic pressure leading to shunt retraction out of peritoneal cavity. Previous publication shows importance of location of insertion of peritoneal shunt affects retraction rate with lower half of abdominal wall having higher rates of retraction.

Main limitations of this study are the small patient sample and the retrospective analysis, constraining the interpretation and conclusions that can be drawn. Additionally, we acknowledge the lack of systematic positioning for pressure readings for all patients. Pressure readings were collected in varied position, making comparison more difficult. The correlation between thecal pressure in different positions and its association with intracranial pressure is not yet completely identified. Future studies should ensure systematic positioning for all pressure readings. It would be of further interest to add different manoeuvres (e.g. Valsalva to increase abdominal pressure) whilst assessing thecal pressure.

This study offers novel insight into telemetric sensor use in the lumbar theca, for patients avoiding cranial surgery on medical, personal or occupational grounds. The cases described showcase the variety of patients for which lumbar thecal telemetry would be useful. Thecal pressure monitoring can provide a wealth of information in a field which is very limited at present. The clinical significance of this could be drawn to measure changes in pressure rather than discreet values, however further research is required in this area.

## Funding

Not applicable.

## Conflicts of interests

Not applicable.

## Availability of data and material

Not applicable.

## Code availability

Not applicable.

## Ethics approval

Not applicable.

## Consent for publication

Not applicable.

## List of abbreviations

CSF: cerebrospinal fluid
ICP: intracranial pressure
LP: lumboperitoneal
VP: ventriculoperitoneal
IIH: idiopathic intracranial hypertension
NPH: normal pressure hydrocephalus
AMV: arteriovenous malformation
